# Compensation of Hysteresis on Piezoelectric Actuators Based on Tripartite PI Model

**DOI:** 10.3390/mi9020044

**Published:** 2018-01-26

**Authors:** Dong An, Haodong Li, Ying Xu, Lixiu Zhang

**Affiliations:** College of Mechanical Engineering, Shenyang Jianzhu University, Hunnan East Road No.9, Hunnan New District, Shenyang 110168, China; lihaodong@stu.sjzu.edu.cn (H.L.); yxu@sypi.com.cn (Y.X.)

**Keywords:** piezoelectric actuators, hysteresis nonlinearity, Prandtl–Ishlinskii (PI) model, hysteresis compensation, micropolarization

## Abstract

Piezoelectric ceramic actuators have been widely used in nanopositioning applications owing to their fast response, high stiffness, and ability to generate large forces. However, the existence of nonlinearities such as hysteresis can greatly deteriorate the accuracy of the manipulation, even causing instability of the whole system. In this article, we have explained the causes of hysteresis based on the micropolarization theory and proposed a piezoelectric ceramic deformation speed law. For this, we analyzed the piezoelectric ceramic actuator deformation speed law based on the domain wall theory. Based on this analysis, a three-stage Prandtl–Ishlinskii (PI) model (hereafter referred to as tripartite PI model) was designed and implemented. According to the piezoelectric ceramic deformation speed law, this model makes separate local PI models in different parts of piezoelectric ceramics’ hysteresis curve. The weighting values and threshold values of the tripartite PI model were obtained through a quadratic programming optimization algorithm. Compared to the classical PI model, the tripartite PI model can describe the asymmetry of hysteresis curves more accurately. A tripartite PI inverse controller, PI inverse controller, and Preisach inverse controller were used to compensate for the piezoelectric ceramic actuator in the experiment. The experimental results show that the inclusion of the PI inverse controller and the Preisach inverse controller improved the tracking performance of the tripartite PI inverse model by more than 80%.

## 1. Introduction

**Figure 1:**
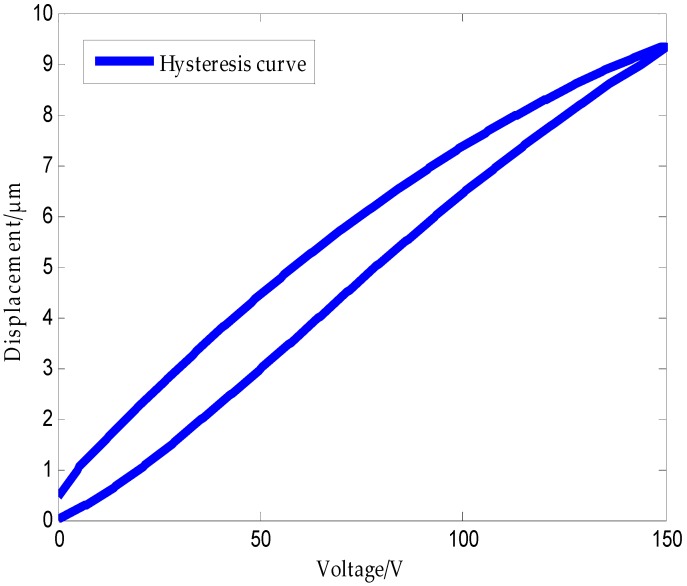
Hysteresis characteristic of a piezoelectric ceramic.

In recent years, the rapid development of ultraprecision machining technology has led to higher positioning accuracy standards of the micropositioning platform driven by some functional materials such as piezoelectric ceramics. Piezoelectric ceramics actuators (PCAs) have been widely used in precision positioning applications, such as scanning and microscopic technologies [1,2], micromanipulators [3], atomic force microscopes [4,5,6], and ultraprecision machine tools [7,8]. This is because of their ability to achieve high precision and versatility to be implemented over a wide range of applications [9]. However, the existence of hysteresis in PCAs often limits the operation performance of the actuators. Therefore, it is highly desirable to compensate for the hysteresis so that the piezoelectric devices can have a virtually linear relationship, or one-to-one mapping between the control signal and the output displacement [10]. Figure 1 presents the relationship between the displacement and the voltage across a piezoelectric actuator. It can be seen from the figure that when the voltage is applied across the piezoelectric ceramic, the step-up displacement curve does not coincide with the step-down displacement curve, and the displacement does not return to zero after the applied voltage is reduced to zero. This phenomenon is called the piezoelectric ceramic hysteresis phenomenon. This means that the actuator output displacement depends not only on the input or applied voltage at the present time, but also on the input history [11]. The intrinsic nonlinear and multivalued hysteresis in the piezoelectric actuator has the potential to cause an inaccuracy in, or even instability of, its applied system. The maximum error resulting from the hysteresis can be as much as 10–15% of the path covered [12]. It is obvious from the above analysis that the available approaches for the identification of piezoelectric-actuated stages containing the hysteresis and linear dynamics are still an open problem [13]. Therefore, establishing a precise control model of piezoelectric ceramics, and (based on this model) controlling the hysteresis nonlinearity of piezoelectric ceramics so as to improve the control precision of piezoelectric ceramics, has become a hot issue discussed by many scholars, globally [14,15].

As increasingly more researchers focus on PCAs, there have been numerous attempts to use models to compensate for hysteresis. These are broadly divisible into two, namely, physics-based models (white/gray box) and phenomenological models (black box) [16]. The physics-based models are derived from the physical means of the hysteresis and can be strictly verified. The physical model refers to a scientific concept that is abstracted out from a large number of experiments for the convenience of research, excluding secondary factors and highlighting the main factors. One of the advantages of physics-based models is their clear physical meaning. However, due to the complicated form, physics-based models are not commonly used in the control of PCAs [17]. The commonly used hysteresis models based on the hysteresis nonlinearity of piezoelectric ceramics are the Jiles–Atherton (J–A) model [18] and the Maxwell model [19]. Malczyk et al. proposed an extension of the J–A model, as the J–A magnetic hysteresis model, to describe the hysteresis curve narrowing phenomenon in ferrite ZnMn material. Their new model permits the inclusion of a wide variety of additional effects observed in ferromagnetic materials without invalidating the well known and broadly used J–A model parameters. Experiments prove the feasibility of this method [20]. Liu et al. presented a Maxwell model to describe the hysteresis in a piezoelectric actuator. They studied the effect of the number of elements and presented both the forward and inverse algorithms. Further, they used the inverse Maxwell model and obtained almost linear performances of the hysteresis compensation. The results of their experiment validate the effectiveness of the proposed algorithm and showed a reduction in hysteresis nonlinearity from 13.8 to 0.4% [21]. Phenomenon-based models are the ones in which researchers generalize and summarize input and output data and the phenomena of practical experiments, utilizing mathematical methods to directly build a mathematical model to satisfy the experiment rule regardless of the physical meaning, such as the Preisach model [22], the Prandtle–Ishlinskii (PI) model [23], the Duhem model [24], and the Bouc–Wen model. Song et al. proposed a novel modified Preisach model to identify and simulate the hysteresis phenomenon observed in a piezoelectric stack actuator. Their approach can handle a varying-frequency dependence by employing a time-derivative correction technique. Parameter estimation and model verification demonstrated high accuracy of the derived model, keeping the deviation in a low percentage range (about 2–3%) [16]. Lin et al. reformulated the Bouc–Wen model, the Dahl model and the Duhem model as a generalized Duhem model to compare the performances of variant hysteresis models with respect to the tracking reference. Since the Duhem model includes both the electrical and mechanical domains, it has a smaller modeling error compared to the other two hysteresis models. Finally, a real-time experiment confirmed the feasibility of their proposed method [25]. Wang et al. proposed a novel modified Bouc–Wen (MBW) model to describe the asymmetric hysteresis of a piezoelectric actuator. They used a polynomial-based non-lag component to realize the asymmetric hysteresis property. The results demonstrate that their model is superior to its competitors’ models in describing the asymmetric hysteresis of a piezoelectric actuator [26]. However, the lack of a physical meaning makes the above-mentioned model difficult to understand. Simultaneously, none of the abovementioned models reveal the cause of hysteresis from a microscopic point of view, thus, modeling errors in these modeling methods are inevitable.

In this study, we first analyze the causes of the hysteresis based on the micropolarization mechanism. Then, by observing the hysteresis curve of piezoelectric ceramic and establishing the deformation speed law of piezoelectric ceramics, we explain the deformation rate of piezoelectric ceramics at different stages, making use of the nucleation rate of microscopic domain evolution. After that, according to the proposed piezoelectric ceramic deformation speed law, we split and then recombined the play operator, and the improved PI model is proposed. Finally, the improved PI model is compared with the traditional PI model and Preisach model. The experimental results show that the accuracy of the improved PI model is increased by more than 80% as compared to the traditional PI model and Preisach model.

This paper is organized as follows: Section 2 describes hysteresis based on the microscopic polarization mechanism and domain wall theory, and reveals the cause of hysteresis from the microscopic point of view. A novel piezoelectric ceramic deformation speed law is proposed, and its analysis presented in Section 3. Section 4 presents the proposed tripartite PI model based on the deformation rate law of piezoelectric ceramics. A contrast experiment with traditional PI model and Preisach model is presented in Section 5. Finally, Section 6 provides a summary of discussion and future works.

## 2. Causes of Hysteresis

### 2.1. Micromechanism

The piezoelectric ceramics are obtained from ferroelectric ceramics after the polarization treatment, and thus, the property of piezoelectric ceramics is consistent with those of ferroelectric piezoelectric dielectric materials. Under the influence of an electric field, they have electrostriction effect, inverse piezoelectric effect, and ferroelectric effect [27].

The electrostriction effect is caused by dielectric polarization. In the presence of an electric field, dielectric molecules get polarized, thereby generating dielectric stress and the corresponding deformation. However, due to the strong mutual attraction between the nucleus and the electrons, the applied electric field is not sufficient to destroy the dielectric property; moreover, compared with the piezoelectric effect, the electrostrictive coefficient is several orders of magnitude smaller than the piezoelectric coefficient; hence, the electrostriction effect is extremely weak in the macro performance [28], and therefore, the output displacement of the piezoelectric ceramic can be ignored.

**Figure 2:**
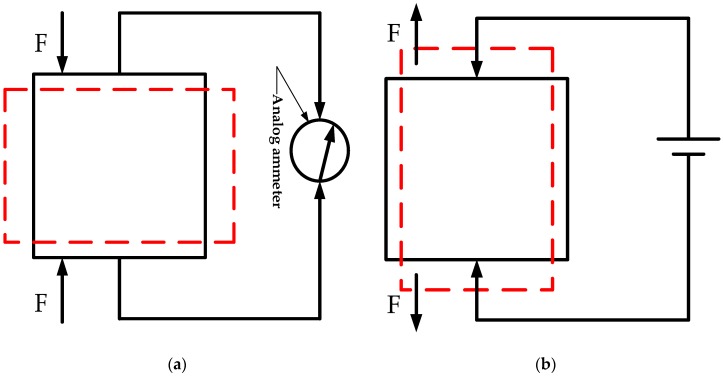
Piezoelectric effect diagram (Red dashed lines indicate after deformation): (**a**) Direct piezoelectric effect diagram; (**b**) Inverse piezoelectric effect diagram. The black rectangle represents the original shape of the piezoelectric ceramic block, and the red dashed rectangle represents the deformed shape.

Curie brothers while studying quartz crystals in 1880 detected crystal deformation [29]. Under the effect of an external force, the surface of the crystal will have polarized charges when a mechanical force is applied. This appearance of electrical polarization is called direct piezoelectric effect, as shown in Figure 2a. On the contrary, if an electric field is applied to the piezoelectric crystal, the crystal not only produces polarization, but also produces deformation. This phenomenon of deformation caused by the electric field is called the inverse piezoelectric effect, as shown in Figure 2b. Piezoelectric ceramic output displacement feature is due to the inverse piezoelectric effect. In general, inverse piezoelectric effect can be expressed as

(1) S=dE

where S is the strain due to the electric field, d is the piezoelectric constant, and E is the applied electric field strength. The inverse piezoelectric effect can be deduced from the above equation, is linear, and there are no hysteresis characteristics.

**Figure 3:**
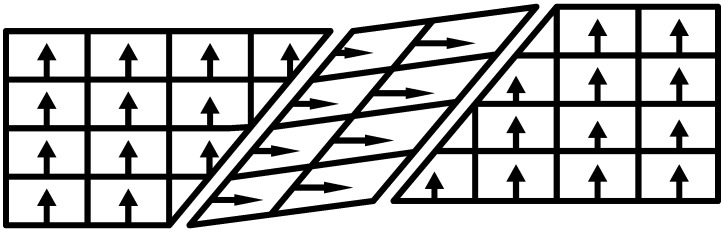
Piezoelectric crystal domain diagram.

Piezoelectric ceramic is a kind of ferroelectric material. Inside the piezoelectric ceramic, in the presence of an external force, the intrinsic dipole moments of the unit cell are arranged neatly in the same direction and cause the piezoelectric ceramic crystal to be in a highly polarized state. Spontaneous polarization in ferroelectric materials always splits into a series of small regions with different polarization directions, so that the electric fields established by spontaneous polarization with the external space offset each other. Therefore, the entire single crystal is nonelectrical. These small areas with the same direction of spontaneous polarization are called domains. There are usually four directions inside a piezoelectric ceramic transducer: the 71° domain, the 90° domain (as shown in Figure 3), the 109° domain, and the 180° domain. It should be noted that, for the crystal strain, only a non-180° domain steering contributes to the displacement of the PCAs, while a 180° domain steering has no effect on the volume effect [30]. Spontaneous polarization of the domain will reorient under the influence of an external electric field. This phenomenon of reorientation of the spontaneous polarization in a piezoelectric ceramic in the presence of an external electric field is known as the ferroelectric effect.

**Figure 4:**
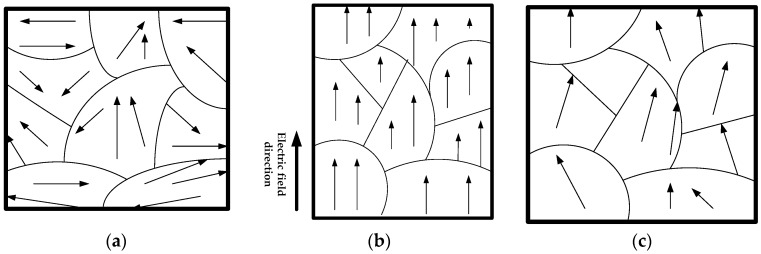
Schematic diagram of the spontaneous polarization alignment: (**a**) before; (**b**) during; (**c**) after presence of an electric field.

Therefore, we define the micropolarization mechanism of a piezoelectric ceramic as if the direction of the applied electric field in a piezoelectric ceramic is the same as the polarization direction. Then, the domain inside the piezoelectric ceramic will have a certain degree of steering and elongation and the boundary of the domain will also produce elongation deformation. Therefore, the piezoelectric ceramic will have an elongation deformation along the polarization direction (as shown in Figure 4).

### 2.2. Analysis of the Causes of Hysteresis

When the applied electric field strength exceeds a certain critical field strength (the field strength that begins to turn the electric domain), the strain of the piezoelectric ceramic (except for the inverse piezoelectric effect) occurs, thus steering the non-180° domain (which is not completely reversible) and gradually starts to dominate. When the field strength is on the decline, some non-180° domains cannot be restored to the same level as at the time of increasing field strength.

In this study, we assume that *N*1 is the number of unit cells making non-180° domain turns in the piezoelectric ceramic when the field strength is increased and *N*2 is the number of unit cells making non-180° domain turns in the piezoelectric ceramic when the field strength is reduced. From the above analysis, we can conclude that N1>N2; this partially irreversible non-180° domain causes the hysteresis in the displacement of the PCAs. Furthermore, the greater the field strength, the more irreversible the non-180° domain, and greater the hysteresis displacement of the PCAs.

## 3. Piezoelectric Ceramic Deformation Speed Law

### 3.1. Derivation of Deformation Speed Law

**Figure 5:**
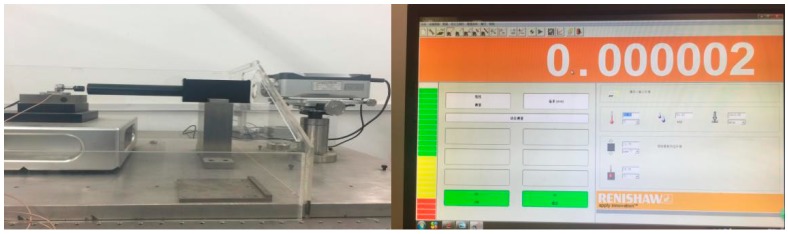
XL-80 laser interferometer ((Renishaw plc, Gloucestershire, UK)).

**Figure 6:**
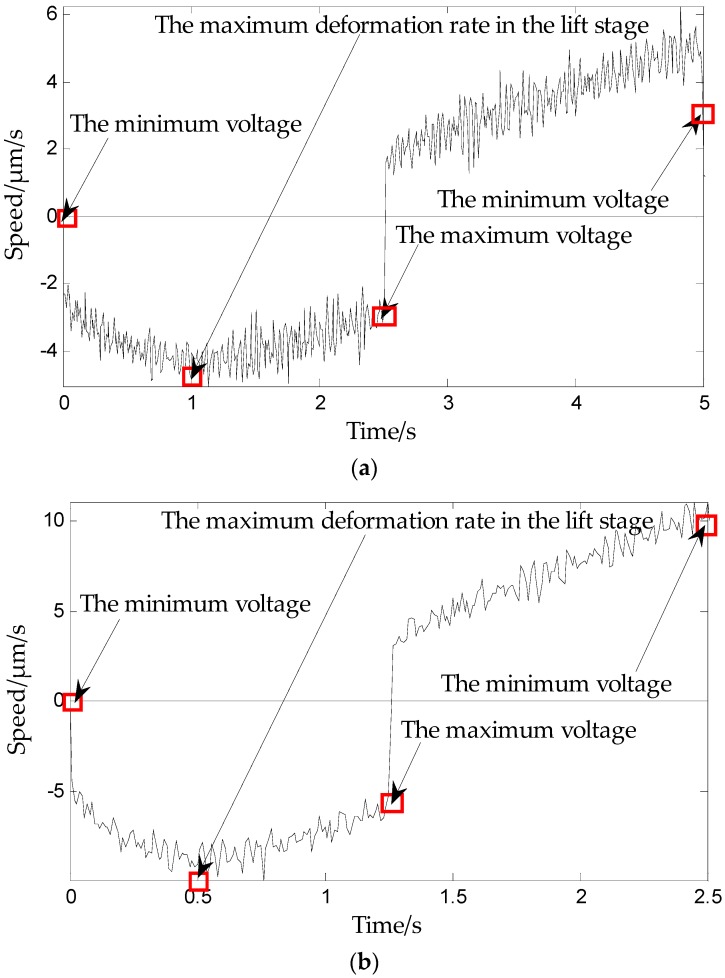

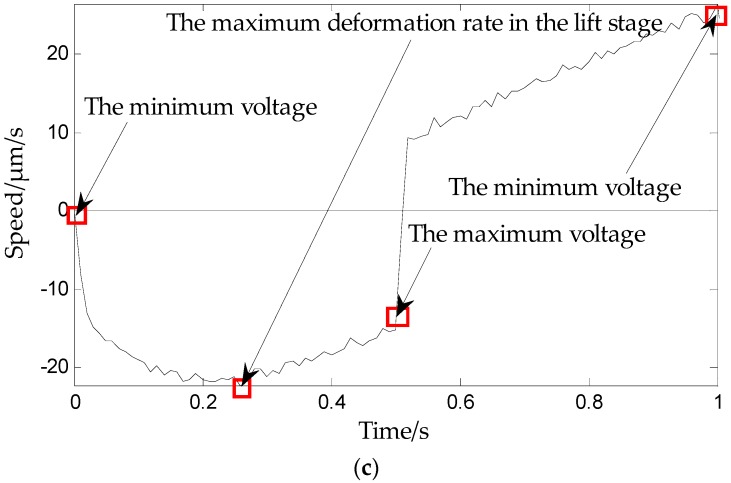
Deformation rate of piezoelectric actuators at an applied triangle wave voltage of 150 V and a frequency of (**a**) 0.2 Hz; (**b**) 0.4 Hz; (**c**) 1 Hz. Below the timeline, the voltage is loaded from the minimum voltage (0 V) to the maximum voltage (150 V). Above the timeline, the voltage drops from the maximum voltage (150 V) to the minimum voltage (0 V).

**Figure 7:**
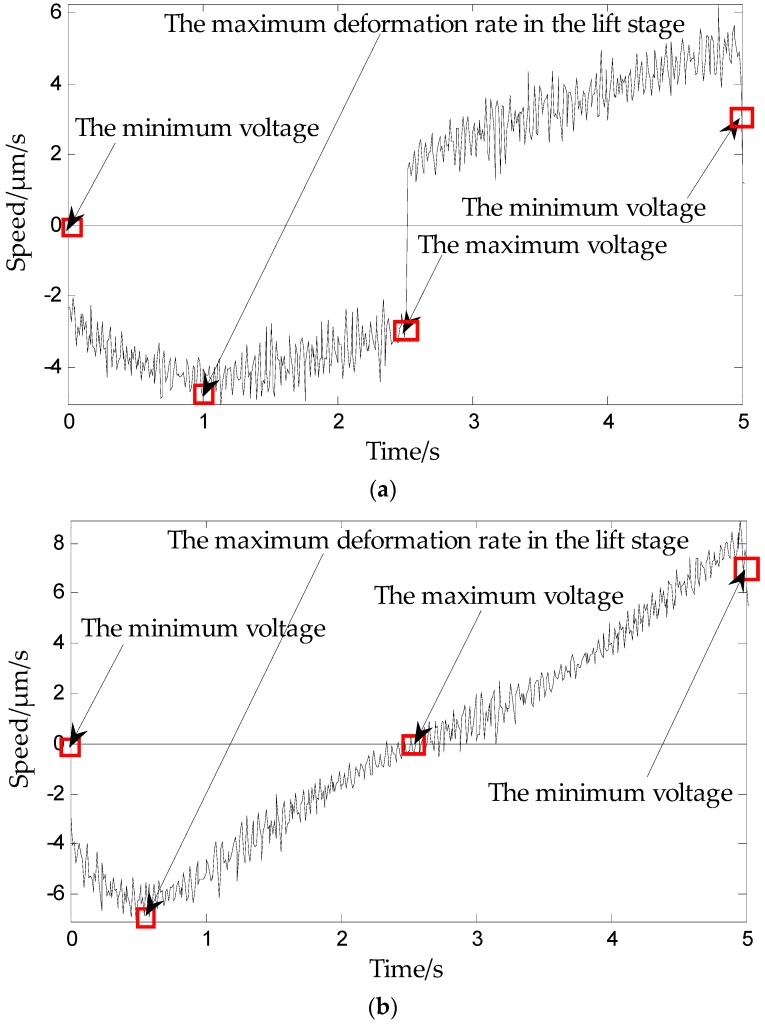

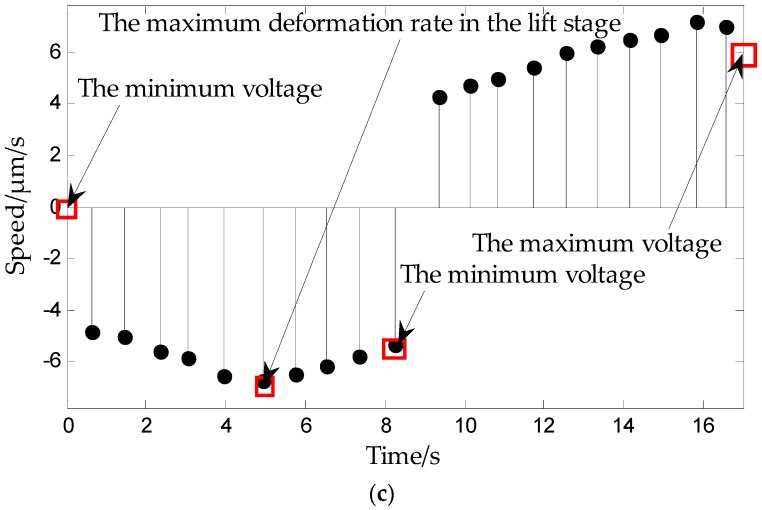
Deformation rate of piezoelectric actuators for an applied voltage of 150 V with frequency 1 Hz in (**a**) triangular wave form; (**b**) sign-wave form, *u* = 150(sinπ*t*/5) positive half cycle; (**c**) Manually added, 0 V–150 V–0 V, at steps of 15 V. Below the timeline, the voltage is loaded from the minimum voltage (0 V) to the maximum voltage (150 V). Above the timeline, the voltage drops from the maximum voltage (150 V) to the minimum voltage (0 V).

We utilized the Renishaw XL-80 (Renishaw plc, Gloucestershire, UK) laser interferometer (shown in Figure 5) to measure the deformation rate of piezoelectric ceramics for voltages ranging from 0 V to 150 V. Figure 6 shows the deformation rates of the PCAs for an applied triangle wave voltage of 150 V driven by different frequencies. Figure 7 shows the deformation rates of the PCAs for an applied triangular wave, a sine wave, and a manually added voltage.

As can be seen from Figure 6, although the triangular wave voltage frequency is different, the three sub-plans followed the same law: the deformation rate in the lift stage is below the timeline (which is the elongation rate), showing the trend of first increasing and then decreasing with time, during the boost period. Deformation rate change is not monotonic and the maximum value is taken as shown by the arrow in the figure. The return deformation rate is above the timeline (which is the contraction rate), showing an increasing trend over time: in the voltage reduction phase, deformation rate increases monotonically, with the maximum appearing at the end of voltage reduction phase. Figure 7 shows that although the applied voltage wave forms are different, the three sub-plans follow the above law.

Therefore, we propose the deformation rate law of piezoelectric ceramics: In the phase of voltage increase, the deformation rate of piezoelectric ceramics first increases and then decreases, and there is an inflection point voltage. During the voltage drop phase, the deformation rate of piezoelectric ceramics increases monotonically without the inflection point.

### 3.2. Analysis of Deformation Speed Law

In 2007, Rabe et al. proposed that when an electric domain turns under the influence of an electric field, the entire domain is not oriented like a dipole. Instead, the following four stages occur: new domain nucleation, vertical growth of new domain, horizontal expansion of the new domain, and new domain merger [31].

In this study, we analyze the above law based on the nucleation rate of microscopic domain evolution. As already mentioned in Section 2.1, piezoelectric ceramic deformation is due to the internal electric domain steering. Experiments of predecessors have confirmed that the physical mechanism of electrical domain inversion is the nucleation process and the nucleation rate of the domain is a function of the applied electric field [32]. Hence, through the change of nucleation rate, one can obtain the domain inversion volume change rate. Merz et al. obtained the relationship between the new domain nucleation rate and the applied load through experiments, generally conducted in the lower electric field range (*E* = 0.1 kV/cm–1.0 kV/cm). They found the nucleation rate in line with the exponential relationship [33]

(2) $$n_{1}=k_{1}\exp(\frac{-\delta }{E})$$

In the higher electric field range (*E* > 1.0 kV/cm), the nucleation rate conforms to the power function

(3) $$n_{2}=k_{2}E^{1.4}$$

where *n*_1_, *n*_2_ represent the numbers of nucleations per unit time per unit area, *δ* is the activation of the electric field, and *k*_1_, *k*_2_ are constants.

We assume that the electric field changes uniformly. Taking saturated electric field as 2*E_c_*, the total number of domains contained in the piezoelectric ceramic is

(4) $$N=\int_{0}^{E_{c}}k_{1}\exp(\frac{-\delta }{E})dE+\int_{E_{c}}^{2E_{c}}k_{2}E^{1.4}dE$$

In a certain electric field, flipped domains are only related to the applied electric field. Therefore, the deformation rate of piezoelectric ceramics at low electric field strengths can be expressed as

(5) $$\alpha_{1}=\frac{\int_{0}^{E}k_{1}\exp(\frac{-\delta }{E})dE}{\int_{0}^{E_{c}}k_{1}\exp(\frac{-\delta }{E})dE+\int_{E_{c}}^{2E_{c}}k_{2}E^{1.4}dE}$$

At high electric field strengths, the deformation rate can be expressed as

(6) $$\alpha_{2}=\frac{\int_{0}^{E_{c}}k_{1}\exp(\frac{-\delta }{E})dE+\int_{E_{c}}^{E}k_{2}E^{1.4}dE}{\int_{0}^{E_{c}}k_{1}\exp(\frac{-\delta }{E})dE+\int_{E_{c}}^{2E_{c}}k_{2}E^{1.4}dE}$$

We define *E*_0_, *E*_1_, *E*_2_, *E*_3_…, *E_n_*_-1_, *E_n_* as the field strength points at equal intervals on the axis of coordinates, while the distance between the two adjacent pressure points is defined as *h_i_* = *E_i_* − *E_i_*_−1_. Assuming that *E_c_* is an inflection point electric field (can be considered as a constant), and using the geometric meaning of definite integral, we can get

(7) $$\alpha_{1}=\frac{\sum_{i=1}^{c}h_{i}\exp(\frac{-\delta }{E})}{M}$$

In the formula $M=\int_{0}^{E_{c}}k_{1}\exp(\frac{-\delta }{E})dE+\int_{E_{c}}^{2E_{c}}k_{2}E^{1.4}dE$, since *E_c_* is a constant, *M* can also be considered as a constant. Similarly,

(8) $$\alpha_{2}=\frac{m+\sum_{i=c}^{n}h_{i}k_{2}E^{1.4}}{M}$$

where $m=\int_{0}^{E_{c}}k_{1}\exp(\frac{-\delta }{E})dE$ is also a constant.

As can be seen from Equations (7) and (8), *α*_1_ and *α*_2_ are electric field functions. However, the exponential function grows much faster than the power function. Therefore, the growth rate of *α*_1_ is obviously greater than the growth rate of *α*_2_, which means that the deformation rate of the piezoelectric ceramics in the range 0~*E_c_* is greater than the deformation rate of piezoelectric ceramics in the range *E_c_*~2*E_c_*. In the piezoelectric deformation curve, the deformation speed of the voltage rise phase first increases and then decreases, and there is an inflection point of deformation rate. Combining this with the applied voltage period, we can determine the piezoelectric ceramic hysteresis curve inflection point voltage.

Based on this basic fact, this study proposes a tripartite PI model—a modeling method for the hysteresis characteristics curves of piezoelectric ceramics.

## 4. Hysteresis Modeling

**Figure 8:**
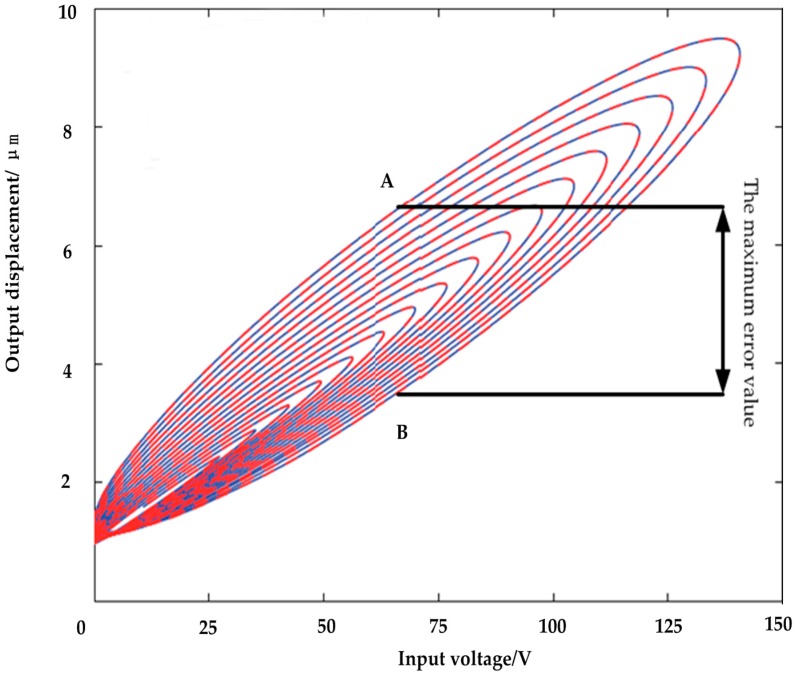
Piezoelectric ceramic input-output hysteresis curve.

Whether in scanning tunneling microscopes, atomic force microscopes, or other precision positioning systems, quick and accurate positioning of the probe is desired. However, due to hysteresis, it is difficult for the probes to locate the correct position quickly and accurately. Various ways to reduce errors and improve the positioning accuracy are currently in practice. In this paper, we discuss the use of modeling methods, as shown in Figure 8.

From the piezoelectric ceramic hysteresis curve shown in Figure 8, it can be seen that every time the voltage rises and reduces, a hysteresis error is introduced; however, the maximum error occurs in the main hysteresis loop (the distance from A to B in Figure 8). Therefore, in this study, we only model the main hysteresis loop of the hysteresis curve. As a next step, we will conduct a study on the remaining displacements of the hysteresis curve.

### 4.1. Play Operator and Prandtle–Ishlinskii Model

**Figure 9:**
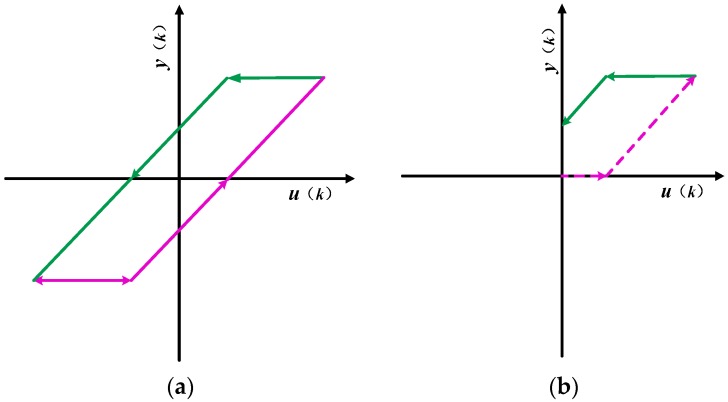
(**a**) Complete play operator; (**b**) play operator in practical application.

The PI hysteresis model can be thought of as consisting of a stack of hysteresis operators. The mathematical expression for the play hysteresis operator shown in Figure 9a is

(9) y(k)=max{u(k)−r,min[u(k)+r,y(k−1)]}

where *k* is the input time, *r* is the threshold of the play operator, *u*(*k*) is the input of the operator, and *y*(*k*) is the output of the operator. The initial value of hysteresis operator is defined as

(10) $$y\left(0\right)=\max\left\{u\left(0\right)-r,\min\left[u\left(0\right)+r,h_{0}\right]\right\}$$

If the piezoelectric actuator is started from the power off state, the value of *h*_0_ is 0.

The PI hysteresis model was established in 1970 by the Russian mathematician Krasnoselskii, developed from the Preisach model and it was referred to as the PI model. It is formed by different play operators with different thresholds. The play operator is similar to the hysteresis curve in shape. Different play operators are multiplied by different weighting values and superimposed on one another to obtain the piezoelectric ceramic hysteresis PI model. The mathematical expression for the PI model is

(11) $$Y\left(k\right)=\sum_{i=1}^{n}w_{i}\times y_{i}\left(k\right)=\sum_{i=1}^{n}w_{i}\times \max\left\{u\left(k\right)-r_{i},\min\left[u\left(k\right)+r_{i},y\left(k-1\right)\right]\right\}$$

where *w_i_* is the weight of each hysteresis operator in the mathematical sense, *n* is the number of operators, *Y*(*k*) is the output of the model at the moment *k*, and *r_i_* is the threshold of the hysteresis operator. The vector form of Equation (11) is

(12) $$Y\left(k\right)=w^{T}\times y\left(k\right)$$

where the threshold vector is *W* = (*w*_1_,···, *w_i_*,···, *w_n_*)*^T^*, the state vector of the operator at the moment *k* is *y*(*k*) = (*y*_1_(*k*),···, *y_i_*(*k*),···, *y_n_*(*k*))*^T^*, and the state vector of the operator at initial time is *y*(0) = (*y*_1_(0),···, *y_i_*(0),···, *y_n_*(0))*^T^*.

In the actual experiment, the step-down phase of the standard play operator is only partially present in the first quadrant, leading to its limited accuracy in describing the backhaul part of the hysteresis curve. The input voltage is always positive, which is increased from 0 V, therefore, in actual modeling, only a portion of the standard play operator is used. The input and output of the unilateral play operator are completely cuffed in the first quadrant, as shown in Figure 9b. The dotted and solid lines shown in Figure 9b are the output of the operator during increasing and decreasing voltage times. For *u*(*k*) ≤ *r*, the output *y*(*k*) always remains zero. For an input *r* ≤ *u*(*k*) ≤ *u*_max_, the operator output is *u*(*k*) − *r*. When the input voltage drops from peak *u*_max_ to *u*_max_ − 2*r*, the operator output is *u*_max_ − *r*. After this, the operator output *y*(*k*) is *u*(*k*) + *r*, until the voltage drops to zero. The output of the operator whose threshold *r* ≥ 0.5*u*_max_ does not have an output of *u*(*k*) + *r*.

### 4.2. Traditional PI Modeling and Inverse Model

We define *d*(*k*) as the output displacement that corresponds to the input voltage of the piezoelectric ceramic at time k; the expression of the error signal *e*(*k*) at time *k* is

(13) $$e\left(k\right)=d\left(k\right)-Y\left(k\right)=d\left(k\right)-w^{T}\times y\left(k\right)=d\left(k\right)-y^{T}\left(k\right)\times w$$

The square of the error *e*(*k*) is

(14) $$e^{2}\left(k\right)=d^{2}\left(k\right)-2d\left(k\right)y^{T}\left(k\right)w+w^{T}y\left(k\right)y^{T}\left(k\right)w$$

In this study, the accuracy of modeling is measured by the addition of the squared errors $\sum_{k=1}^{n}e^{2}\left(k\right)$, where *n* is the number of sampling points. The weight vector *w* of the objective function is obtained from the quadratic programming algorithm, i.e.,

(15) $$\begin{array}{l} f\left(w\right)=\sum_{k=1}^{n}e^{2}=\sum_{k=1}^{n}{\left[d(k)-y^{T}(k)\right]}^{2}\\ =\sum_{k=1}^{n}d^{2}\left(k\right)-2\sum_{k=1}^{n}d\left(k\right)y^{T}\left(k\right)w+\sum_{k=1}^{n}w^{T}y\left(k\right)y^{T}\left(k\right)w \end{array}$$

The cross-correlation function row vector $R_{xd}^{T}$ and autocorrelation function matrix $R_{xx}$ are defined as

(16) $$R_{xd}^{T}=\sum_{k=1}^{n}d(k)y^{T}(k)$$

(17) $$R_{xx}=\sum_{k=1}^{n}y(k)y^{T}(k)$$

Equation (15) can now be expressed as

(18) $$f\left(w\right)=\sum_{k=1}^{n}d^{2}(k)-2R_{xd}^{T}w+wR_{xx}w^{T}$$

This indicates that *f*(*w*) is a quadratic function of the weight coefficient vector *w*, which is an upwardly concave parabolic surface and a function with a unique minimum. The weight coefficient is adjusted so that *f*(*w*) is the minimum, i.e., we find the minimum drop along the curved surface corresponding to the parabolic path. Here, we use the gradient descent method to find this minimum.

For Equation (18), we take a derivative with respect to the weight coefficient *w*, and we obtain the gradient of *f*(*w*) as

(19) $$\begin{array}{l} \nabla (k)=\nabla f(w)\\ =-2R_{xd}+2R_{xx}w \end{array}$$

Make ∇(k) = 0, and the optimal weight coefficient vector can be obtained.

(20) $$w=R_{xx}^{-1}R_{xd}$$

In order to ensure the positive definite of the quadratic matrix, the principle of threshold selection is $r_{\max}<u_{\max}$, $r_{i}=\frac{i}{n}\max\left|u\left(k\right)\right|,i=0,1,2,\cdots ,n-1$. Using a program written in MATLAB software (R2017b, MathWorks, Inc., Natick, MA, USA) for the operation, the parameters of the traditional PI model are obtained. They are tabulated in Table 1.

**Figure 10:**
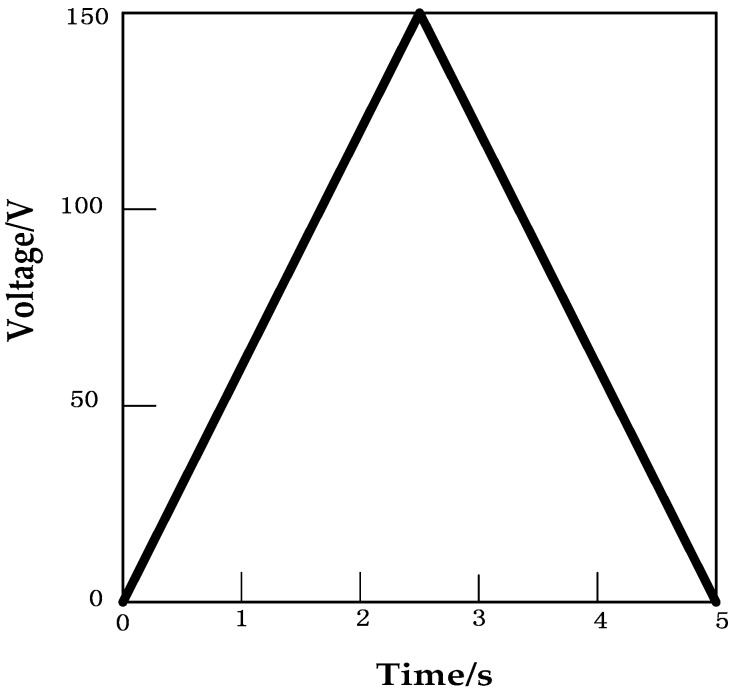
Input voltage of the piezoelectric ceramic actuator.

**Figure 11:**
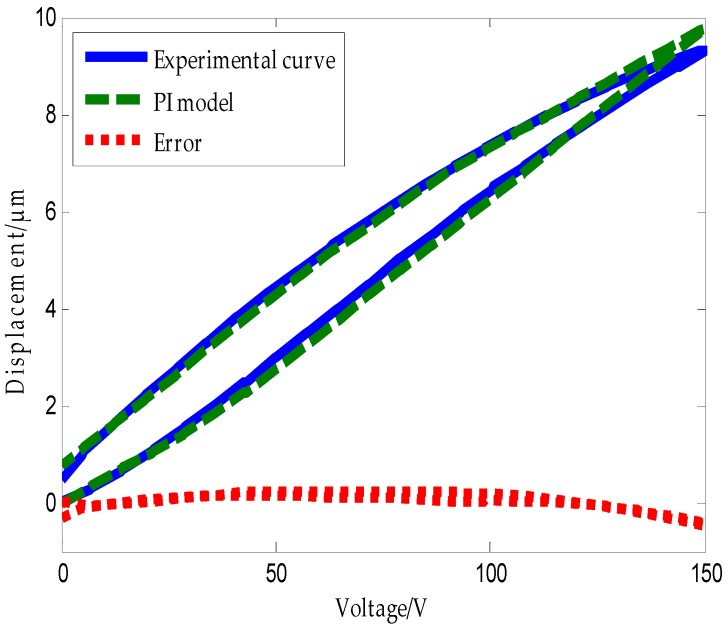
Hysteresis curve of piezoelectric ceramic actuators and PI model.

The triangular wave form shown in Figure 10 was applied to the piezoelectric ceramic. The experimental hysteresis curve of the piezoelectric ceramic and the curve obtained using the traditional PI model along with the error plot are shown in Figure 11. Error analysis using MATLAB software shows that the mean absolute error of the traditional PI model is $\delta_{1}=\frac{1}{n}\sum_{k=1}^{n}\left|e_{k}\right|=0.13947\;\mu m$.

The inverse PI model is also a PI model. The threshold vector and the weight vector of the PI inverse model can be calculated using the relationship between the PI model and its inverse model. The PI model has an analytical inverse, $r_{i}^{\prime }=\sum_{j=1}^{i}w_{j}\left(r_{i}-r_{j}\right)$, i=1,⋯,n; $w_{1}^{\prime }=\frac{1}{w_{1}}$, $w_{i}^{\prime }=-w_{i}/\left[\left(\sum_{j=1}^{i}w_{j}\right)\left(\sum_{j=1}^{i-1}w_{j}\right)\right]$, i=2,⋯,n; $u_{i}\left[0\right]=\sum_{j=1}^{i-1}w_{j}y_{i}\left[0\right]+\sum_{j=i}^{n}w_{j}y_{j}\left[0\right]$, i=2,⋯,n. Hence, the output expression of the PI inverse model at the time *k* is

(21) $$U\left(k\right)=\sum_{i=1}^{n}w_{i}^{\prime }\times u_{i}\left(k\right)=\sum_{i=1}^{n}w_{i}^{\prime }\times \max\left\{y\left(k\right)-r_{i}^{\prime },\min\left[y\left(k\right)+r_{i}^{\prime },u\left(k-1\right)\right]\right\}$$

**Figure 12:**
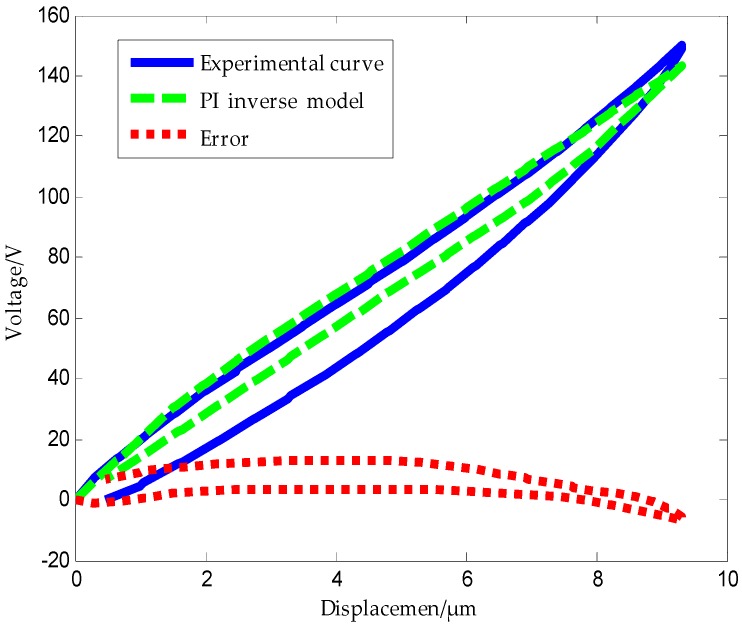
Hysteresis curve of PI inverse model.

Figure 12 shows the experimental hysteresis curves of the piezoelectric ceramic and the ones obtained using the PI inverse model along with the error plot.

Error analysis using MATLAB software shows that the mean absolute error of the traditional PI inverse model is $\delta_{2}=\frac{1}{n}\sum_{k=1}^{n}\left|e_{k}\right|=0.29435\;\mu m$. From the above analysis, we can conclude that the traditional PI model and its inverse model exhibit large modeling errors.

### 4.3. Tripartite PI Model Based on the Deformation Rate of Piezoelectric Ceramics

**Figure 13:**
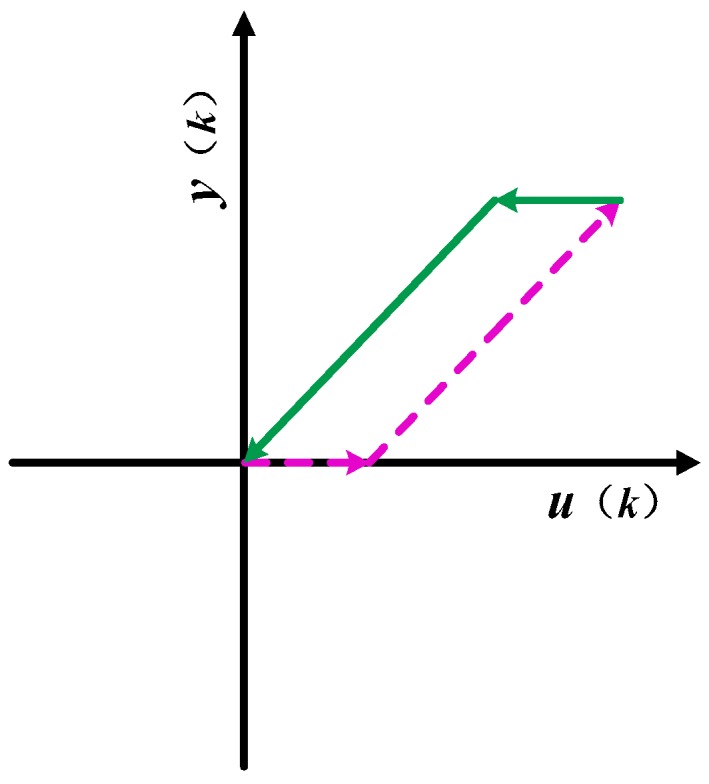
Schematic of one side play operator.

In Section 3.1, we obtained the piezoelectric ceramic deformation speed law, and here, we propose a tripartite PI modeling method based on this law. As already mentioned in Section 4.1, the step-down phase of the standard play operator is only partially present in the first quadrant, therefore, the standard play operator has a limited description of the backhaul of the hysteresis curve. The operator used in the tripartite PI modeling method is a unilateral play operator. The input and output of the unilateral play operator are completely limited to the first quadrant, as shown in Figure 13.

The output expression of unilateral play operator is

(22) $$\left\{ \begin{array}{l} y\left(0\right)=\max\left\{u\left(0\right)-r,\min\left[u\left(0\right),0\right]\right\}\\ y\left(k\right)=\max\left\{u\left(k\right)-r,\min\left[u\left(k\right),y\left(k-1\right)\right]\right\} \end{array}\right.$$

The dashed part is the boost part and the solid part is the buck part. The model is based on the theory presented in Section 3. A data collection experiment of the piezoelectric ceramic hysteresis curve under the triangle wave pressure provides support for modeling. The inflection point is captured based on Figure 6 and Figure 7, which show the maximum deformation speed in the rising process.

Modeling steps:

(1) The selection of operators is based on the principles of concave-convex consistency, which means that in the hysteresis curve, the concave and convex parts of the curve correspond to the boost part and the depressurization part of the play operator, respectively.
(2) The rising curve rises from zero voltage to the inflection point voltage *u_if_* (*u_if_* refers to the voltage indicated by the arrows in Figure 6 and Figure 7), i.e., when the deformation speed rises from 0 to the maximum. The relationship between the voltage and displacement is described by a single lateral play operator as shown in Figure 13 (the dotted portion).
(3) The rising curve rises from the inflection voltage *u_if_* to maximum voltage *u*_max_ (*u*_max_ refers to the maximum point voltage applied to the piezoelectric ceramic during the whole rising cycle. It is 150 V here). Voltage–position relation in this part is described by a single lateral play operator as shown in Figure 13 (the solid line). One side play operators and hysteresis curves have a counter clock directivity. The reducing portion and rising process in the second part manifest the epirelief characteristic. The reducing portion of play operators point to the origin of coordinates while the second rising hysteresis curve deviates from it. Therefore, we need to model in reverse when we use play operators in the reducing part to describe the second rising process of the hysteresis curve.
(4) The retraced curve’s relation that reduces from the maximum to zero voltage is described by a single lateral play operator as shown in Figure 13 (the solid line).

**Figure 14:**
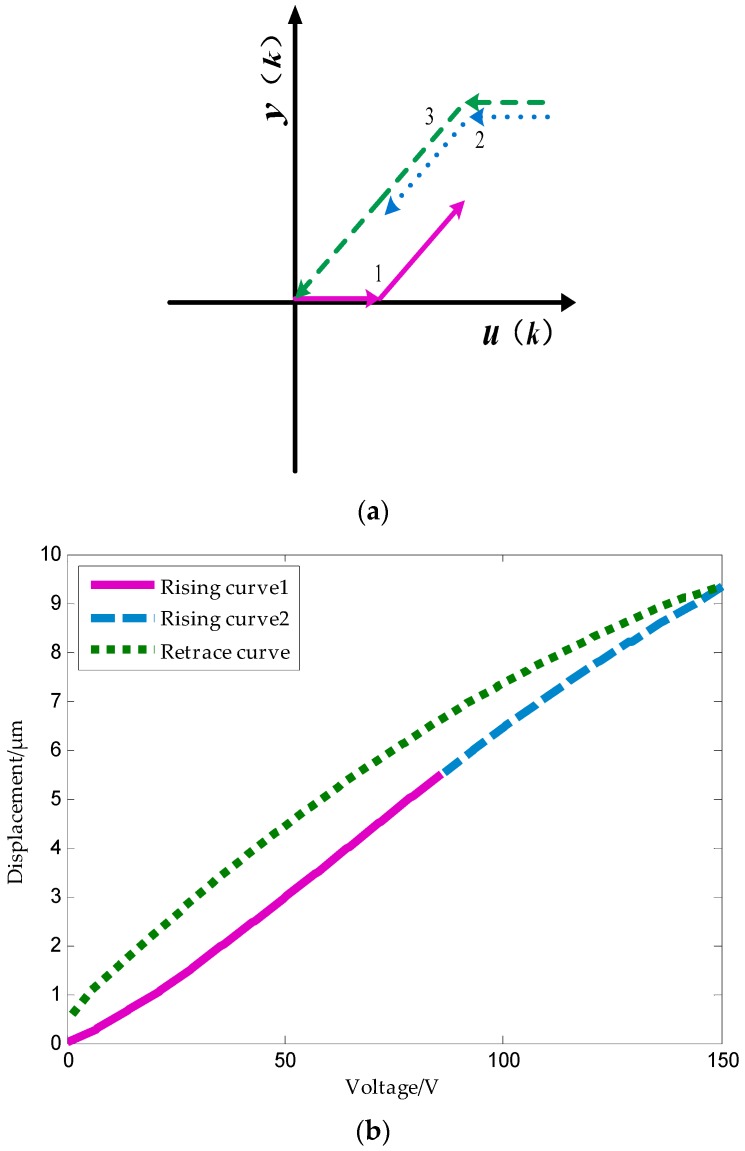
Relation of the play operator and the hysteresis curve.

The application of operators in respective parts of the hysteresis curves during the model process are shown in Figure 14.

The weight identification algorithm and threshold selection principle are consistent with Section 4.2. Table 2 shows the identification parameters of the tripartite PI model.

**Figure 15:**
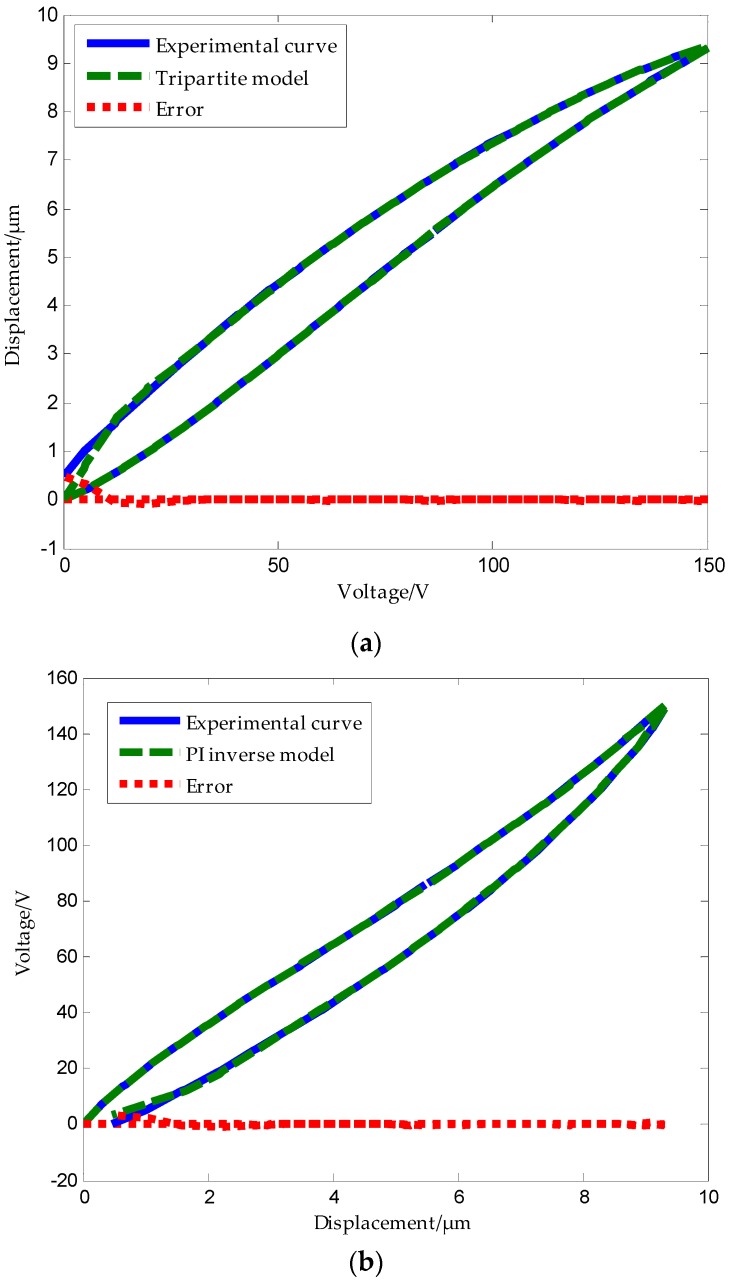
The tripartite PI model and its inverse model: (**a**) experimental and tripartite PI model hysteresis curves of a piezoelectric ceramic actuator; (**b**) experimental and tripartite PI inverse model hysteresis curves of a piezoelectric ceramic actuator.

The tripartite PI model also includes the tripartite PI inverse model. Figure 15a,b show the modeling result of the tripartite PI model and its inverse model, respectively. Through MATLAB software modeling, we obtained the mean absolute error of the tripartite PI model as $\delta_{3}=\frac{1}{n}\sum_{k=1}^{n}\left|e_{k}\right|=0.02137\;\mu m$.

It should be noted that the unilateral play operator used for modeling returns to the origin when the voltage comes back to zero, and the displacement of the tripartite PI model obtained by the weighted addition of the unilateral play operator is also forced to zero when the voltage drops to zero. Since the actual hysteresis curve does not return to zero when the voltage drops to zero, the tripartite PI model shows a high error when the voltage is close to 0 only during the process of reducing pressure.

## 5. Experiment Results and Discussion

**Figure 16:**
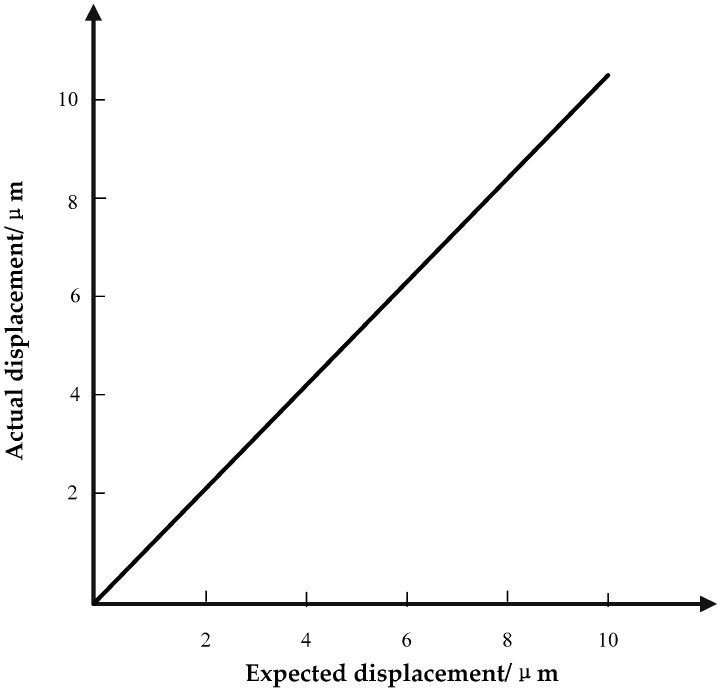
Desired relation of expected displacement and actual displacement.

The purpose of the microdisplacement positioning system is to make the expected piezoelectric ceramic output displacement equal to its actual displacement, as shown in Figure 16.

**Figure 17:**
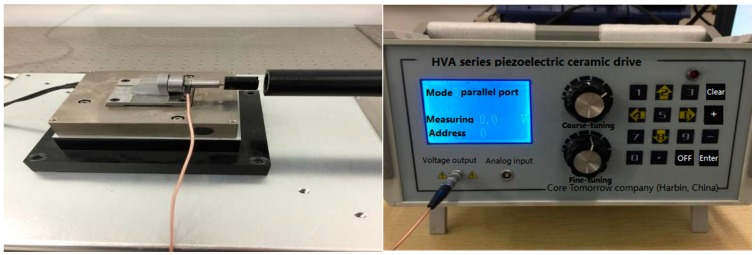
Experimental setup.

**Figure 18:**
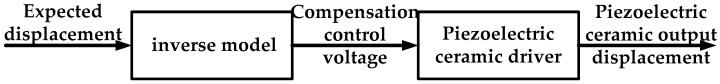
Block diagram of PI inverse model.

Hence, in order to verify the effect of tripartite PI inverse model on the piezoelectric ceramic driver’s hysteresis compensation, we designed a piezoelectric ceramic hysteresis model control effect comparison experiment. The PCA used for this study was the PSt150/4/7VS9 piezoelectric ceramic made by Core Tomorrow company (Harbin, China). We used the driving power HVA-150D. A3, made by Harbin Core Tomorrow Science & Technology Co., Ltd., to generate driving power (the input voltage was in the range of 0 to 150 V and the output displacement was in the range of 0 μm to 9.5 μm) to drive the piezoelectric ceramic and a Renishaw XL-80 laser interferometer to measure the displacement. The equipment used for the experiment was as shown in Figure 17**.** The driving power communicated with the host computer through the standard parallel port (SPP) parallel communication port. Here, we refer to the Preisach model parameters used in Song et al.’s study [16] and we used the experimental data in this study to establish the PCAs Preisach model. The desired displacement was taken as the input of the PI inverse model, the Preisach inverse model, and the tripartite PI inverse model. Thus, we got three sets of control voltages. These three sets of voltages were used to control the piezoelectric ceramic via the driving power. The output displacement was collected and recorded in real time by the laser interferometer. The control block diagram is shown in Figure 18. After the experiment, the data was processed and compared.

**Figure 19:**
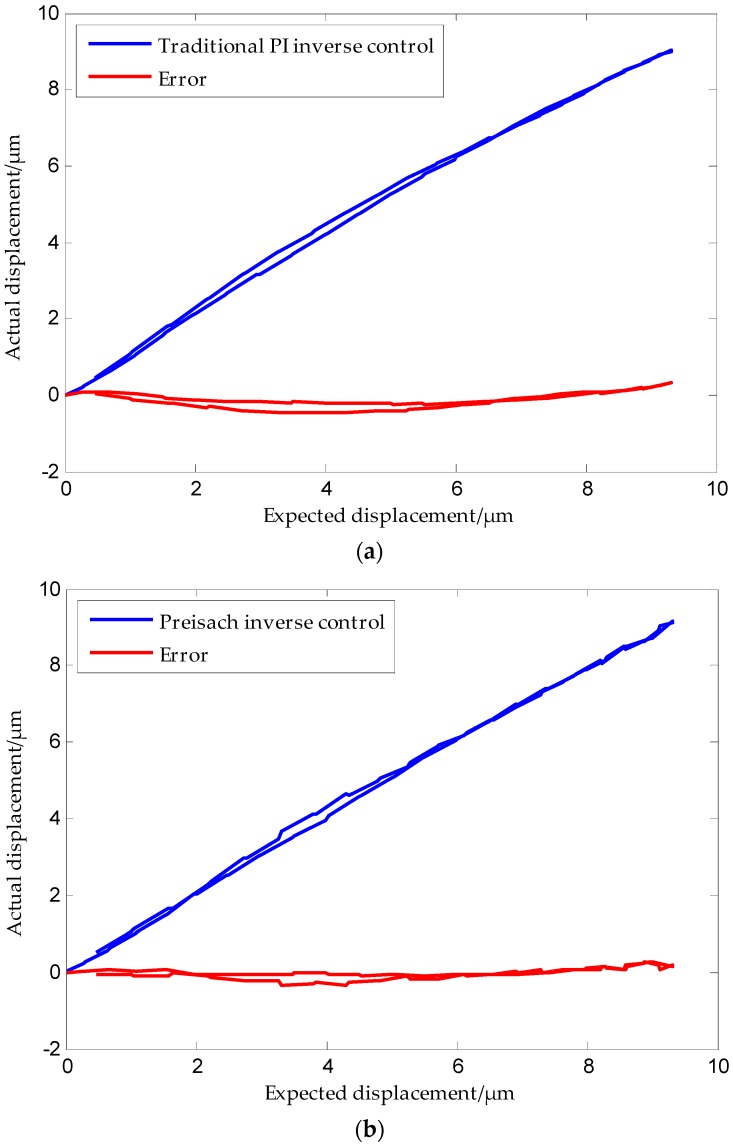

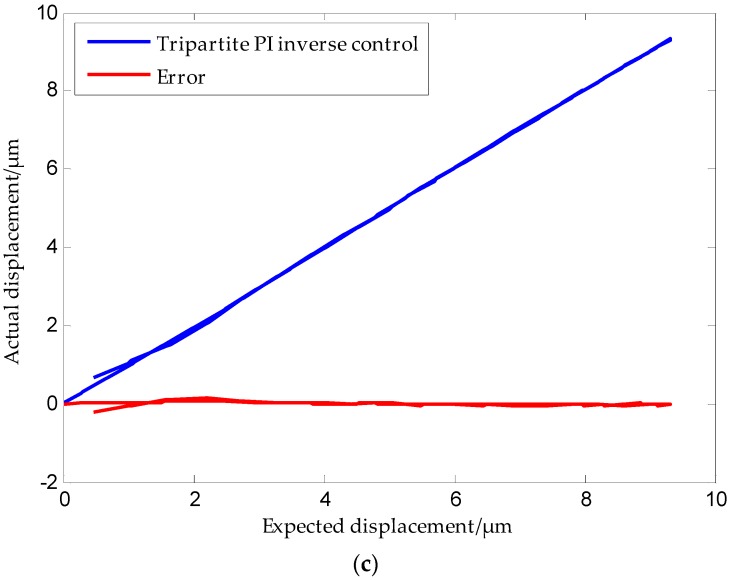
Positioning accuracies of three kinds of inverse models: (**a**) PI inverse model; (**b**) Preisach inverse model; (**c**) Tripartite PI inverse model.

The results obtained are shown in Figure 19. The mean absolute errors (*MAE*) of the traditional inverse PI model, the Preisach inverse model, and the tripartite PI inverse model compensation controllers are *MAE* = 0.19019 μm, *MAE* = 0.10893 μm, and *MAE* = 0.03549 μm, respectively.

**Figure 20:**
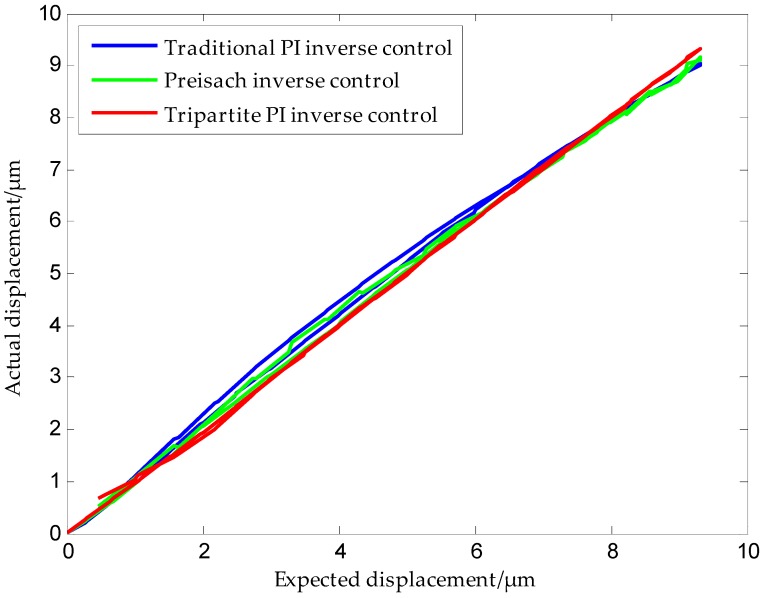
Comparison of the positioning accuracies of different models.

Figure 20 shows a comparison of the positioning accuracies of the three models. It can be seen from the figure that the positioning accuracy of the tripartite PI model was higher than that of the traditional PI model and the Preisach model. Error analysis shows that the positioning accuracy was improved by more than 80% in the case of the tripartite inverse model when compared to the other two models. Experiments confirm that the proposed modeling method was effective.

## 6. Conclusions

In this study, through the observation of the deformation rate for piezoelectric ceramics during the process of applying voltage, we arrived at the general law of the deformation rate of piezoelectric ceramics and this law has a certain universality. The tripartite PI model is proposed on the basis of the deformation rate law. The hysteresis curves of piezoelectric ceramics with different deformation rate laws are modeled to obtain the tripartite PI inverse model. Since the second segment is inverted, in the actual control application, the control voltage of the second stage needs to be used in reverse order. The tripartite PI modeling method does not introduce other parameters and other operators. The model is simple and easy to construct, and can accurately describe the characteristics of the main hysteresis loop. The tripartite PI inverse model has even more accurate precision as a series controller in the micro process and is used in the reverse cycle. Next, we will conduct a study about the remanent displacement of the hysteresis curve to broaden the scope of application of the model.

## Figures and Tables

**Table 1:** Parameters of the Prandtl–Ishlinskii (PI) model. (*i* is the number of sampling point, *r_i_* is the threshold of the play operator, *w_i_* is the weight of each hysteresis operator in the mathematical sense).

*i*	*r_i_*	*w_i_*
1	0	0.0493
2	15	0.0298
3	30	0.0120
4	45	0.0090
5	60	0
6	75	0
7	90	0
8	105	0
9	120	0
10	135	0

**Table 2:** Parameters of tripartite PI model. (*i* is the number of sampling point, *r*_1_ is the threshold of the first stage play operator, *w*_1_ is the weight of the first stage hysteresis operator in the mathematical sense; *r*_2_ is the threshold of the second stage play operator, *w*_2_ is the weight of the second stage hysteresis operator in the mathematical sense; *r*_3_ is the threshold of the first stage play operator, *w*_3_ is the weight of the first stage hysteresis operator in the mathematical sense).

*i*	*r*_1_	*w*_1_	*r*_2_	*w*_2_	*r*_3_	*w*_3_
1	0	0.0415	0	0.0529	0	0.0322
2	6.42	0.0097	15	0.0027	15	0.0081
3	12.84	0.0082	30	0.0067	30	0.0054
4	19.26	0.0067	45	0.0022	45	0.0044
5	25.68	0.0051	60	0	60	0.0065
6	32.10	0.0043	75	0	75	0.0039
7	38.52	0.0026	90	0	90	0.0087
8	44.94	0.0016	105	0	105	0.0016
9	51.36	0	120	0	120	0.0009
10	57.78	0	135	0	135	0.0008
